# Anæsthetics

**Published:** 1865

**Authors:** Julius Chesebrough

**Affiliations:** Professor of Mechanical Dentistry in the Ohio Dental College, 1865


### ANÆSTHETICS.

### *Their Properties and Constituents, and mode of Administration.*

BY JULIUS CHESEBROUGH, D. D. S., PROFESSOR OF MECHANICAL DENTISTRY IN THE OHIO DENTAL COLLEGE, 1865.

[Read before the Mississippi Valley Dental Association, Feb. 22, 1865, at Cin., O.]

The human system is indeed a complicated one. In that of no other is there even a near approach to it, with its framework of bone, superstructure of muscle, and those fine interwoven connexions of arteries and veins, with nervous subtleties that bring all parts in one common bond, and give the whole that power which makes man superior to every other being, that is not supreme. The study of all these parts is one of great length, and the mind that will even know the rudiments of the depth of knowledge to be acquired to be thoroughly conversant must spend years of patient toil. It has been truly said that the more we study and know of the great complications of earth, air, fire, water, the elements, and humanity, the less we know.

We know that the brain is the great source from whence spring those tendrils of subtlity, which, through their intricate connexions with all parts of the human system, render man more than a mere machine. These tendrils habiting every tissue and cell of tissue become acquainted with every abnormal condition of a part, and that is at once conveyed to the fountain head,—the throne of reason. To undertake to explain the manner in which this knowledge of an affected part is transmitted from the part through all the intricacy of the network of nerves that form the nervous system, would absorb too much time and would be a digression from the object of this paper. Still it is somewhat necessary that we should understand the general principles of nervous action that we may be conversant with, and know how we are acted upon by, those subtle agents, whereof it is my province to speak. We all know that we have a brain, a combination of organs, vast in itself, intricate in its anatomy, and incomprehensible as to its functions and actions. Proceeding from this great being (for the brain is the embodiment of being) is the spinal cord, and radiating from the spinal cord are the various nerves that supply the whole system. Four of the senses, hearing, seeing, smelling and tasting, are in direct communication with the brain itself. That of feeling throws its impression upon the brain through the spinal cord. This then we may consider the great main avenue of sense; receiving and giving out, from the extremes of the system and supplying the internal arrangement of the system with power to act. Every spinal nerve has at its origin two parts, divisible, and yet as one,—the anterior or motor, and posterior or sensitive. At a short distance from the origin, these too roots form a knot or ganglion, and their fibres then lay closely along side of each other exchanging fibres to form plexuses where they are associated in the supply of a separate region, (as the lower extremities,) and finally terminating in loops, and anastamosing plexuses. So we see how intimately connected are the two great nerve powers, and what a perfect blending of purpose is maintained by them.

The office of the sensitive root and fibre is to convey to the brain that knowledge of the morbidity of a certain part, and also to convey to the motor, which affects the movement of all parts of the system, that intelligence which will enable it to act according to the desired end.

Indeed man is truly and wonderfully made, and we can comprehend the vast power and sublimity of the Almighty, who out of the dust created man after His own image.

The sensitive root of the nerve, is that one which is susceptible of receiving an impression, and thus enabling it to act. Were there no danger of the human system ever being deranged, there would be no need for sensitive nerves. But such is not the case. Though the mechanism which makes man was pronounced perfect, yet he takes not the proper care of that mechanism, and suffers it to accident through his carelesslessless.

What is the result of that carelessness? It is a derangement of the mechanism, and that derangement results in what is termed pain.

Thus we find that *pain* is a derangement of the sensitive fibres of the nerves through neglect or carelessness of this great system by its occupant.

Were our system in perfect accord, we should have no pain, and the fact of the existence of pain, in *any* part, shows us that there is a great derangement *in* that part, or one in sympathy with it. Thus we become aware that pain is the greatest of all bodily evils, and the torture of it is beyond the power of mortal to withstand. It sometimes however, becomes necessary to be inflicted with pain for our own good, that we should suffer to relieve. That when a part is in an abnormal condition, it may be necessary to undergo an infliction of pain to relieve that part, or to render it again normal.

The stoutest heart quails and shudders at the dread of an infliction of pain, and there are but few that can withstand the slightest pain. Of the surgeons knife, or the dentists forceps, every one has an unmitigated horror. Of the exquisite torture of surgery, and that "hell of pains" of the dentist, we may all well stand in dread. There is no one who has not experienced the presence of this dire monster—pain,—and how to baffle his subtlity and power well deserves our attention, and our study.

It was well known to the ancients that there were drugs potent enough to avert pain or to render the human system incapable of a realization of it. Among these comprising many whose effect was narcotic, or capable of throwing a person into a deep sleep; and while in that state, operations have been performed, some of them capital in their extent. But we find no extended record of the use of drugs as a means to avert pain, nor do we at the present day find mention of them among that class of agents which we call anæsthetics.

This name is given to that class of agents which have the power to suppress the sensitive action of the nerve, so as to deprive the body of that element which we denominate pain.

I have said that the ancients used drugs as anæsthetic agents, but not these alone. We find mention made of the fact that a "pot of coal burned in a close room, will sometimes put one to sleep, when drugs failed; but great care must be taken else the patient dies." Also, "that when one has been subjected to the fumes of burning coal, all sense has been lost, and it is hard to wake them, even by resorting to harsh measures," and in one case, "a large abscess was opened while the person was under sleep." Thus we find that the "fumes of coal" nothing else than carbonic acid gas, was known to be an anæsthetic agent. This agent acts upon the system as a life destroyer; not as a suspender of vitality. It produces suffocation, and as the sensitive fibres are the ones which first receive the impression and convey it to other parts, they are of course the first ones to succumb, and assume, as it were, death. As a general applicant to produce anæsthesia, this substance should never be used, though there are many cases where with benefit it might be brought into requisition, as it can be made to produce a deep sleep, when other methods have failed, but the closest watch must be kept upon the patient, and when once dropping into its embrace, it must at once be removed, and pure air given. The effect will be lasting enough to give the worn out patient a sleep that will be of great benefit.

But we must look further into the arena to bring out the agent that will enter into all the complications of the system, and hold them in close embrace, and freedom from sensibility.

These substances are to be classed under the heads of local and general anæsthesia. Local is that which, by being applied directly to the part, produces there a non-susceptibility of pain. Of these I may only speak of two or three: Congealations, or the application of a freezing mixture, and the application of aconite, either its tincture or extract, and electricity. Of the freezing, or application of intense cold, little can be said in its favor. Generally operations which require the use of an anæsthetic agent are so deep within the structure or are so far from the surface, that to apply such an agent, would but be the total destruction of the part, and thus complicate by making matters worse, rather than better. For superficial operations it might do, but even then it is not advisable. Aconite acts from its powerful element to narcotise the part with which it is brought in contact, rather than to promote an anæsthetic influence. It is sometimes used applied upon cotton, to the gums over roots of teeth with benefit, but it is not sure in its action, and all patients are not alike in their capacity of susceptibility. Electricity has often been used for minor operations, and cases are reported where capital operations have been performed, but the records are few and far between. The effect is to paralize the sensitive roots and fibres of the nerves. It does not affect the brain, or the action of the heart, simply neutralizing the power which the sensory fibres have over the motor; as we can see by the contractions which take place when this agent is applied. The manner of using it is for the patient to be connected with one pole of a battery, and the other attached to the knife, if a surgical operation is performed, to the forceps if a dental. By bringing the knife or forceps upon the part there is immediately formed an electro-current and the paralyzing power at once begins. Still we find it not much in use, being of more benefit to play upon the imagination than of real utility.

We will now consider those agents which are comprised under the name of general anæsthitics. These comprise chemical combinations known under the general names of chloroform, ether, nitrous oxide gas, and alcohol.

The properties of this latter however, enter into the combination of the two former, that we must consider it worthy of our attention. Though not a powerful anæsthetic, instances have been known whereby inhaling the vapor of alcohol has brought the patient under a soothing influence, which was anæsthetic in its nature. Deprive it however of the equivalent of water which it contains and we have a powerful anæsthetic. Thus it may be made to assume a form potent and all powerful. The constituents of alcohol $C^4$, $H^6$, $O^2$, we find similar to that of ether $C^4$, $H^5$, $O^1$. ether being nothing more or less than alcohol deprived of its equivalent of water. We find that the effect of inhalation of alcohol vapor is very much the same as when it is taken internally. At first there is that lightness of the whole system, that makes the body feel as of airy rather than earthly matter; then the quick following revolutions of the brain, and a seeming turning round of everything. All the world moves and soon our motor nerves succumb to the influence and we have presence of mind enough left to prostrate ourselves, before we have that giddiness which will surely prostrate us. Now the subtle agent begins to reign supreme, and we are folded within its grasp, powerless; to move, we can not; to speak, only in disarticulations, mutterings, not within the mind's comprehension; all seems lost to us and our care is little for either ourselves or the outside world. Still there is a comprehension, though in a low state; and in some persons this even is lost, and all is in obscurity. Upon such persons as these last, the anæsthetic has control enough at this time to perform any operation. Still the power of telling at once, who of our patients are susceptible of the effect of alcoholic stimulus, and who are not, is not given us, and we therefore labor under that disadvantage. Therefore must we give up this agent and search about for one that will accomplish the desired end. Sulphuric ether, an agent first brought to the notice of the profession in 1846, as an anæsthetic, seems to have some, if not all the desired effects, which are found lacking in alcohol. Deprived of (from alcohol) an equivalent of each, hydrogen and oxygen give the carbon, that subtle agent in all general anæsthetics, free scope to work. In its administration, we find that probably the best method of giving it is to form a piece of stiff paper into a cone; at the apex of the cone have an opening an inch in diameter, and around the base have a napkin secured to the paper. Let the diameter of the cone at the base be about the width of the patient's face. Now place upon the napkin about a half ounce of ether, and place the cone over the patient's face. The best position of the patient is a horizontal one, as the circulation is impeded less in its ebb and flow while the patient is in this position than any other. Besides this, a horizontal one is that in which the surgeon operates. Direct your patient now to be perfectly composed, and take a deep inspiration. The orifice in the apex will supply atmospheric air in quantity sufficient to enable the ether to effect the system. Atmospheric air is an essential to the safe and proper exhibition of ether and chloroform. Were it not administered in connection with these agents the patient would die of strangulation, or suffocation. Caution your patient to breathe free and easy. And after four or five inhalations have been taken remove the cone for an instant that you may see the indications of the face. This should retain its natural color, heightened, but in no case must there be a bleached look to the skin; at this moment you can notice the breathing which must be free, not with a murmuring or choaking sense; there must be no rattle in the throat or bronchia, if there is, desist at once. With the cone held in one hand let the other be upon the pulse, and note this above all else. It should be nearly as in the natural state, with very little acceleration, and a trifle diminution in volume; but on no account should it assume a rapidity unnatural, or a weakness that is difficult to feel. The great care must be at the start; for if the patient can pass through the first inhalations, and there are no unfavorable symptoms, then they can take it with little risk of ill effect; but if these effects are broken in upon, with heavy breathing, rattling in the bronchia, livid countenance, and rapid pulse, beware!—your patient is nearer death than you are aware. Of the quantity of ether to be administered, there is great diversity of opinion by many who have given it largely. I am inclined to say, that there are no two patients that require the same amount. I have often had full effect produced with less than half an ounce, and I have used a pound without seeming effect. The appearance of the patient must indicate somewhat the extent to which the effect must be carried.

I have noticed that there is always a rigidity of the muscles after a few inhalations have been taken. And that this rigidity occurs at the time when the anæsthetic influence is about being felt. The arm now being lifted falls dead as it is dropped, and a pulling of a few hairs of the head occasion no emotion or action on the part of the patient. For the surgeon, now has arrived the time to cut, and he can go to his work with impunity and a will. The subtleties of etherial air have penetrated the inmost recesses of the body, and the sensory nerves have been overcome; the motors have been wrapped up within the folds of this mystic agent, the brain no longer performs its function, and we have our patient before us sensitively dead. Only the respiration, and the ebb and flow of life's blood, tell us that the form before us has life. Should signs of recurring consciousness appear, the ether must be again applied, and the patient kept fully under its influence. While we have for the surgeon, the proper time to operate, at the rigidity of the muscles, we find that this rigidity closes the mouth against all. Therefore does the dentist labor under the disadvantage. His application and administration must be carried further. His patient must be carried beyond the contraction, and he can not operate until the muscles have lost that power so that the mouth can be readily opened. And it is for this reason that I am inclined to the belief that the majority of deaths that have occurred, in proportion, have been from the administration of anæsthetics for dental operations.

We are compelled to carry our patients further toward the brink of existence, than is the surgeon; and it devolves upon us therefore to know just where that line comes. As the patient comes under the influence of ether or either of the anæsthetic agents, the operator should watch the time narrowly, and at the moment the mouth can be opened, that is the moment to operate. Generally there is need of but one inhalation, for a skillful operator will do all the extracting necessary in the moments the anæsthetic influence is upon the patient. Another disadvantage which the dentist labors under is, that he can not operate upon his patient in a horizontal position. They must have the head inclined or raised so that the teeth can be readily reached.

For this purpose I have a chair in which I can throw a patient in a horizontal position, and when under the anæsthetic influence raise them to a sitting posture; Extract the teeth or operate as I may have occasion, and at once bring them into a horizontal position again, turn them that the blood may run out of the mouth, and let nature do its work to bring them back again to life.

Chloroform needs much the same observation and treatment in its administering. It is a more powerful agent than ether, and therefore should be used with great care. There are slight changes in the pulse and features of the patient; but to describe all these would consume more of your time than I can consent to take. The same directions I have given in relation to ether answer equally well for chloroform, and should be watched very closely.

But I think an effect is obtained better in every respect, more potent than ether, less dangerous than chloroform, when the two agents are combined in the proportion of two of ether to three of chloroform. Seldom do we see any of the ill effects of either agent shown in this combination. It acts quickly and well. The patient feels better after the recovery, and there are none of those after effects which we often see where ether or chloroform have been administered in their pure state.

In many cases we find that neither ether or chloroform or any admixture will bring the patient into an anæsthetic state.

Such conditions are very perplexing in their nature, not to say aggravating.

In such cases I have found that administering alcohol in the same manner as I have directed for the inhalation of either ether or chloroform, has had fine effect when followed by ether or chloroform.

The drinking of a good glass of brandy or whisky has the same effect, and renders the patient susceptible to the effect of chloroform or ether in a few minutes, when before it there was not a sign that denoted any impression being made. In the great majority of cases it has succeeded with me; and I would advise that in nine-tenths of your patients it be administered as a preliminary; in *all* cases where there is great vigor and full vitality. It is entirely inadmissable to administer chloroform or ether when there are pulmonary disorders; and the cases are many upon record where death has ensued from the administration.

Ether and chloroform are powerful agents, and can not be thrown into the hands of an ignorant or unskillful man, without resulting disastrously, and rebounding upon the whole profession. Therefore should we discountenance their use, only by those who have thorough knowledge of their effects, and are capable of using them as the divine power intended they should be used,—with care.

Nitrous oxide gas is the oldest modern form of anæsthetic. The exhilirating properties of this agent have long been known, but not until about 1844 was it known to possess anæsthetic properties. At that time it was used extensively by one or two dentists for the extraction of teeth. It is formed by heating nitrate of ammonia in a glass vessel, passing the gaseous product through an alkaline solution and gathering it in a receiver. Its constituents are as its name indicates: nitrogen and oxygen. Equivalent for equivalent. It is administered by being inhaled into the lungs, *without* any admixture. Thus we see that it is vastly different from ether or chloroform.

At the first breath there seems to be an uplifting of the whole system. At the second there seems to be an expansion of the body to almost unlimited proportions. The third sends the subtle agent to the very extremeties, and there is a tingling of the fingers and toes, and at each successive breath there comes creeping back a benumbing influence, and the patient feels as if he was rapidly sinking. The inhalations at first gentle and easy, now become laborious and stertorious, and but a few moments elapse before a perfect unconsciousness of all bodily pain exists.

The effect at first enlivening and exhilirating now loses itself, and the last impression made upon the mind or perhaps the last word spoken has unlimited sway, and in the brain reigns supreme.

In most cases after the patient has inhaled the gas from a half to a full minute, they will sink into a dreaming state, and there remain until the effect of the gas passes off, (which will be in from one to three minutes), wholly unconscious, not roused by the infliction of the most severe treatment, or that which without the use of an anæsthetic would be unbearable by even the stoutest heart. It would seem as if only the sensory nerves were acted upon.

These only to that effect that entirely obliterate all sense of pain, for the brain seems active and able to comprehend all that is going on about it. Many patients will, on the recovery from the effect of gas, exclaim "I know it all; know all you did; everything you said; and saw every motion, yet was incapable of resistance, and felt no pain."

Its effect upon the system is opposite from that of ether or chloroform.

The pulse is more rapid, and the countenance livid; lips and ears highly colored and as the inhalation proceeds rapidly, changing from red to purple.

A predetermination to resistance on the part of the patient will often complicate matters, as their last impression is to close the mouth.

When such is the case after the effect of the gas passes off or begins to do so, there is little feeling left, or so little that the operation of extracting teeth can be fully carried out even with perfect consciousness on the part of the patient. After consciousness returns, there is no ill feeling, none of those after claps which we frequently see following the administration of ether and chloroform. It can be administered any number of times, as I have seen no more effect noticeable after the twelfth administration than after the first.

Neither have I ever seen the constitution that could not bear it. I have administered it to old and young, given it before giving ether and after it, and I have yet to see the first ill effect from it. In pulmonary diseases it can be given with impunity and with great benefit. Were the province of this paper such, I could give many cases in proof of this fact, but it would be a digression from the intent.

One great benefit to be gained by it, and for which I can advocate it is, that there are none of those scenes enacted that we who have given chloroform and ether are called upon to witness. The patient remains quiet, and with perhaps a motion of the hand as of beating something, is the only manifestation of sense, and all those sensual scenes which have been enacted by those possessed with such high imaginations, we do not see here. The only objection I can see to it is its short duration, the time seldom exceeding one minute till there is full anæsthetic effect, though I have known persons who would be under the full effect for full five minutes. This renders it for general anæsthetic purposes out of question. It can however be administered and the patient kept under the influence for from twenty to thirty minutes. This however has been done but once, and that by a distinguished operator, (Dr. Colton). But for purposes within the province of the dentist, I prefer it above all other agents of which we have knowledge. That it is an excellent anæsthetic, no one who has once seen its administration for a moment doubts.

Now that we have given the general ideas in relation to these subtleties, let us for a few moments look into their formation and see if we can gain any information in regard to their being or the agent that exercises this powerful influence. I have told you that carbonic acid gas is a powerful anæsthetic agent, very often even unto death. We know what the power that exerts *its* influence in this body is; and we find the same agent in alcohol, ether and chloroform. In all these substances carbon forms a great part, alcohol and ether containing each four parts, chloroform two. Both or all of these substances require the admixture of air, in order that their potent effect may be used without fear, for given unadulterated and they would soon suffocate to death. Ether contains four equivalents of carbon, five of hydrogen, and one of oxygen. Carbon affords no more support for respiration or the system than does hydrogen, and the quantity of oxygen is so limited that the life of the being would be soon extinct, consequent upon the protracted inhalation of ether.

Chloroform contains for its constituents three equivalents of chlorine, one of hydrogen, and two of carbon; there being no element which can for a moment support life.

Both these agents have to be mixed with air when administered, requiring, therefore, the oxygen of the air to prevent their inducing suffocation at once. So we are naturally led to the conclusion that the anæsthetic effect is due to the effect of the carbon as the principle, the chlorine and hydrogen of the chloroform and the hydrogen and oxygen of the ether being merely adjunct.

Hydrogen breathed for any length of time produces suffocation without anæsthesia, and chlorine when taken in minute quantities produces spasm of the glottis, and in larger quantities strangulation. That the great element of carbonic acid gas, (carbon) (though suffocation is induced) is the agent by which anæsthesia is produced when carbonic acid acid gas is inhaled, can not admit of a doubt, and if we give the matter that attention that it deserves, we will all arrive at the same conclusion; that the presence of carbon is necessary to produce a perfect anæsthetic state, and that where this agent is lacking, only partial anæsthesia will take place. We find then that perfect anæsthesia is a paralyzation of all nerve power, induced by suffocation, exerted first upon the sensory fibres of the nerves, by them conveyed to the motor fibres and spine, and from the spine to the brain, and that when the effect is felt in that organ, prostration and utter void of sensibility exists. Thus we see how near actual death we approach in the exercise of the ideal. How often have we known this theory and the fact to be brought so near, as both to assume fact. Have not many, if not all of us, had our patient so far gone as to have to administer agents to resuscitate and bring them back to life again. Careful as I have been, it has not been my lot to be free from these ill effects. And the safest and surest agent I have known to restore a patient suffering from the baneful effect of chloroform or ether, is nitrous oxide gas. Now that we have seen what constitutes ether and chloroform, let us look into the composition of nitrous oxide gas and divine, if we can, where its potent agent exists.

I have told you that it was formed of nitrogen and oxygen, equivalent for equivalent. Nitrogen we all know is not a supporter of combustion, and therefore not of life; for no gas that will *not* support combustion will support life. Oxygen on the other hand is the great supporter of combustion and is therefore the life giving element. Pure oxygen when taken into the lungs tends to exalt the whole system, and the effect is a violently rapid living. Had we to breathe pure oxygen we should soon die, from having lived too fast, and it is a wise provision of the deity, that there should be incorporated with oxygen that agent that can control it and render our life a prolonged one. So, as it is with the air we breathe, is it with nitrous oxide gas, the element nitrogen equalizing the oxygen and neutralizing some of its effects. Nitrous oxide gas will support life, but in a rapid state. The whole system becomes rarified by the abundance of oxygen taken into it, and thus the nerves of sense become paralyzed by being acted upon by an agent so much greater and richer in the element of life than they are accustomed to bear. That death will not soon ensue from the prolonged inhalation of nitrous oxide gas, I will not for a moment deny; but when taken in comparison with ether and chloroform, we can but class it as a supporter of life, while these agents are not. Thus we see that the effect of ether and chloroform is brought on by the absence of oxygen, and that all that is necessary to resuscitate our patient will be to give them a sufficient quantity of oxygen to enable the system to throw off the effect of suffocation. This can be done no better than the administering of nitrous oxide gas, and it is surprising and truly wonderful to see how soon the effect is accomplished. Two or three inhalations will generally bring your patient back to consciousness. This I have witnessed myself, when only partial anæsthesia had taken place, and my patient was rapidly sinking. Having nitrous oxide gas in readiness I resorted to that; divining at once that it was oxygen that my patient needed, I placed the tube of my inhaling bag into the mouth and closed the nostrils, and in less time that it has taken to write it, my patient was recovered. In a few minutes I gave that patient nitrous oxide, and extracted the teeth, and she suffered no inconvenience or pain. Can we give a patient chloroform that has diseased lungs, or if we can, dare we give it in any pulmonary disease? Do we not incur great risk in giving it in such cases? Are not the records of death which have taken place, traceable to a greater or less extent to this disease, and the administration of chloroform in that condition? How is it with nitrous oxide gas? Can we give it in cases where there is pulmonary disease? I must from fact say we can. I have several well appointed cases that coroborate this fact. One of a young man who inhaled the gas twice a week and is now well. This was his only treatment. He would take it in and pass into a perfect anæsthetic state, more so than any one else that I have known, and when he recovered from it would say, "Now I feel better, now I breathe easier." I might go on with a recitation of fact, relative to the administration of ether, chloroform and nitrous oxide gas until I tire your patience. My purpose is only to give an insight if possible into the subtle workings of these agents and set us all thinking about this matter. If we do not give it the consideration that belongs to it, we will forever remain in the dark.

We must dig for the root of this matter; what though we tire in the work?

Is it not of importance enough to require our greatest study? Our true mission is to relieve, not inflict, pain, and until we are versed in our calling to enable us to mitigate pain, we bring no credit upon ourselves or our profession.

The study of anæsthetics is one to which we all should devote much time and care. Record our investigations, if we please; they will benefit those who have not advanced as rapidly as we have, and they will be of service to ourselves to show how widely humanity differ.

Begging pardon, if I have overtaxed your time and patience, I submit these remarks as an entering wedge upon the discussion of *Anæsthetics.*

